# E3 ligase LMO7 enhances temozolomide sensitivity by promoting MGMT degradation in lung cancer

**DOI:** 10.1016/j.jbc.2026.111331

**Published:** 2026-02-26

**Authors:** Jiabing Li, Xiaorong Feng, Yunfang Deng, Bei Chen, Lin Miao, Zhaohui Liu, Zhiming Sun, Lei Hu, Jinqiu Ma, Liyuan Zeng, Xiaolong Wang, Yu Zhao

**Affiliations:** 1 The National & Local Joint Engineering Laboratory of Animal Peptide Drug Development, College of Life Sciences, Hunan Normal University, Changsha, Hunan, China; 2 Peptide and Small Molecule Drug R&D Platform, Furong Laboratory, Hunan Normal University, Changsha, Hunan, China

**Keywords:** LMO7, MGMT, E3 ligase, ubiquitination, NSCLC, temozolomide, chemoresistance

## Abstract

Temozolomide (TMZ) is used to treat primary brain tumors and non–small cell lung cancer (NSCLC) brain metastases, yet therapeutic efficacy is often limited by the DNA repair enzyme *O*^6^-methylguanine-DNA methyltransferase (MGMT). Here, we identify the E3 ubiquitin ligase LIM domain only 7 (LMO7) as a regulator of TMZ sensitivity in NSCLC cells by promoting MGMT degradation. LMO7 directly binds MGMT *via* its F-box domain and promotes K48-linked polyubiquitination and proteasomal degradation of MGMT, thereby increasing TMZ sensitivity. TMZ treatment further strengthens the LMO7–MGMT interaction, creating a positive feedback loop that accelerates MGMT degradation and enhances TMZ sensitivity. Consistently, TMZ further strengthened the LMO7–MGMT interaction, and this enhancement was attenuated with the catalytically inactive MGMT-C145A mutant. In patient datasets, lower LMO7 expression and higher MGMT expression correlate with shorter overall survival in lung cancer (Kaplan–Meier Plotter analysis, *p* < 0.01). Together, these results indicate that LMO7-mediated MGMT degradation increases TMZ sensitivity in NSCLC, positioning LMO7 as a potential prognostic biomarker and therapeutic target.

Non–small cell lung cancer (NSCLC) remains a leading cause of cancer mortality worldwide, with an estimated ∼1.8 million deaths annually (1, 2, 3, 4). A particularly devastating complication of advanced NSCLC is the development of brain metastases. In lung adenocarcinoma (LUAD), brain metastases are reported to occur in approximately 40% to 50% of patients over the course of disease progression (5, 6). Intracranial dissemination substantially shortens survival and imposes major neurologic morbidity, whereas the blood–brain barrier further constrains drug delivery and complicates systemic treatment (7, 8, 9). Although platinum-based chemotherapy (*e*.*g*., cisplatin) remains a mainstay for advanced NSCLC, intracranial control is frequently limited, in part because many systemic agents exhibit suboptimal penetration into the central nervous system and resistance emerges during treatment (8, 9, 10, 11, 12, 13). Temozolomide (TMZ), an oral alkylating agent with blood–brain barrier permeability, has therefore been explored for the treatment of NSCLC brain metastases (14, 15, 16, 17, 18). However, clinical benefit is often limited by treatment-associated resistance, which is often associated with elevated *O*^6^-methylguanine-DNA methyltransferase (MGMT) expression across tumor types (17, 19, 20), indicating the need to elucidate the molecular basis of TMZ resistance and to develop strategies that improve TMZ efficacy in NSCLC, particularly in patients with brain metastases.

MGMT is a key regulator of TMZ sensitivity because MGMT directly removes cytotoxic *O*^6^-methylguanine lesions generated during treatment (21, 22). Higher MGMT protein expression has been associated with TMZ resistance in glioblastoma, where TMZ is used as a first-line therapy, and similar associations have been reported in NSCLC (23, 24, 25, 26, 27, 28). Conversely, lower MGMT protein expression correlates with increased TMZ sensitivity and improved clinical benefit (29, 30). Transcriptional regulation of MGMT has been extensively characterized and involves factors including hypoxia-inducible factor 1-alpha, p53, NF-κB, and AP-1 (31, 32). In parallel, MGMT protein level is also regulated by post-translational mechanisms that control protein stability and turnover, including ubiquitin-dependent proteasomal degradation. Several studies suggest that reducing the MGMT protein level can increase TMZ sensitivity (33, 34, 35, 36, 37, 38, 39, 40). Deubiquitinases, such as ubiquitin-specific protease 19 and ubiquitin-specific protease 7, have been reported to stabilize MGMT in glioblastoma (40, 41), whereas E3 ubiquitin ligases mediate substrate recognition and can promote MGMT polyubiquitination and proteasomal degradation. TRIM72 and RAD18 have been reported to promote MGMT degradation in other tumor types (38, 39), consistent with the idea that enhancing MGMT degradation can increase TMZ sensitivity. However, the E3 ubiquitin ligases that target MGMT for degradation in NSCLC remain to be identified.

LIM domain only 7 (LMO7) harbors an F-box domain and has been reported as a component of an SCF-type E3 ubiquitin ligase complex (42, 43, 44, 45, 46, 47). LMO7 displays context-dependent functions in cancer, with both tumor-suppressive and oncogenic activities reported. For example, LMO7 is upregulated in breast cancer (48) and colorectal cancers (49, 50) but is often downregulated in NSCLC tissue relative to normal lung tissue, where LMO7 has been reported to induce apoptosis and to be associated with tumor-suppressive activity (48, 51, 52). Prior work further reports that lower LMO7 levels correlate with decreased cisplatin sensitivity in NSCLC cells (53), consistent with a role for LMO7 in modulating chemotherapy response by regulating protein turnover of DNA repair proteins. Given that increased MGMT protein levels have been associated with resistance to alkylating agents in NSCLC (25, 26, 27, 28), reduced LMO7 expression may contribute to MGMT protein accumulation. Whether LMO7 regulates MGMT protein stability in NSCLC remains to be determined.

In this study, we investigated whether LMO7, an SCF-type E3 ubiquitin ligase component, regulates MGMT protein stability and thereby modulates TMZ sensitivity in NSCLC cells. Mechanistic analyses link the LMO7–MGMT axis to ubiquitin-dependent proteasomal degradation of MGMT, consistent with a post-translational mechanism controlling MGMT protein level in NSCLC. Clinical dataset analyses further indicate that lower LMO7 expression and higher MGMT expression are each associated with shorter overall survival (OS) in lung cancer. Together, these results link an LMO7-dependent ubiquitin pathway to MGMT control and TMZ sensitivity in NSCLC.

## Results

### LMO7 directly binds to MGMT

**Figure 1:**
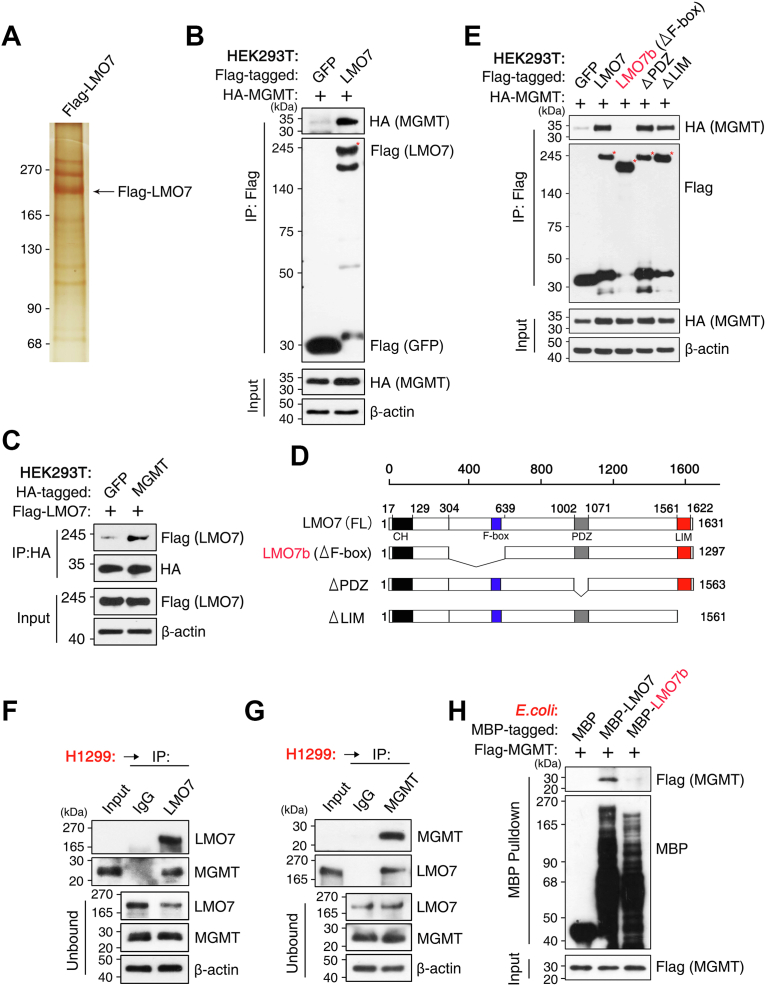
**LMO7 directly binds to MGMT**. *A*, silver staining and LC–MS/MS identification of MGMT as an LMO7-associated protein following affinity purification from H1299 NSCLC cells, with the LMO7 band indicated. *B*, coimmunoprecipitation (co-IP) in HEK293T cells cotransfected with FLAG-LMO7 or FLAG-GFP and HA–MGMT using anti-FLAG M2-agarose beads. *C*, reciprocal co-IP in HEK293T cells cotransfected with HA–MGMT and FLAG-LMO7. *D*, schematic representation of full-length LMO7 and deletion mutants. *E*, mapping analysis showing that the F-box region is required for HA–MGMT interaction. *F* and *G*, endogenous co-IP in H1299 and A549 cells using an anti-LMO7 antibody with IgG control, and immunoprecipitates and unbound fractions (flow-through) were analyzed by Western blotting (WB) to detect endogenous LMO7 and MGMT. *H*, *in vitro* MBP pull-down assay showing direct binding of purified MBP-LMO7 or LMO7b to FLAG-MGMT, with MBP alone as a negative control. *A*, shows a representative silver-stained gel. All other experiments were independently repeated at least three times with similar results. HA, hemagglutinin; LMO7, LIM domain only 7; MBP, maltose-binding protein; MGMT, O6-methylguanine-DNA methyltransferase; NSCLC, non–small cell lung cancer.

To identify proteins associated with LMO7, FLAG affinity purification was performed in H1299 NSCLC cells, followed by silver staining and LC–MS/MS. MGMT was detected among proteins copurified with FLAG-LMO7 (Fig. 1*A*; Table S1). The interaction was validated by coimmunoprecipitation (co-IP) in human embryonic kidney 293T (HEK293T) cells, where FLAG IP retrieved hemagglutinin (HA)–MGMT in the presence of FLAG-LMO7 but not GFP control (Fig. 1*B*), and reciprocal HA IP of HA–MGMT recovered FLAG-LMO7 (Fig. 1*C*). Endogenous co-IP in H1299 cells further supported a native association, as IP with anti-LMO7 antibody recovered endogenous MGMT (Fig. 1*F*) and IP of endogenous MGMT retrieved endogenous LMO7 (Fig. 1*G*). Consistently, endogenous co-IP in A549 cells also detected binding between LMO7 and MGMT (Fig. S1*A*).

To map the LMO7 region required for MGMT binding, LMO7 deletion constructs were generated, including LMO7b lacking the F-box (ΔF-box) and mutants lacking the PDZ or LIM domain (Fig. 1*D*). Co-IP analysis indicated that deletion of the F-box–containing region eliminated MGMT binding, whereas deletion of the PDZ or LIM domain did not abolish the interaction under the tested conditions (Fig. 1*E*). To test direct binding, maltose-binding protein (MBP) pull-down assays were performed with purified recombinant proteins. MBP-LMO7 pulled down FLAG-MGMT, whereas MBP alone and MBP-LMO7b (ΔF-box) did not (Fig. 1*H*). Collectively, these data indicate that LMO7 directly binds MGMT, and the F-box–containing region is required for this interaction.

### LMO7 promotes K48-linked polyubiquitination of MGMT

**Figure 2:**
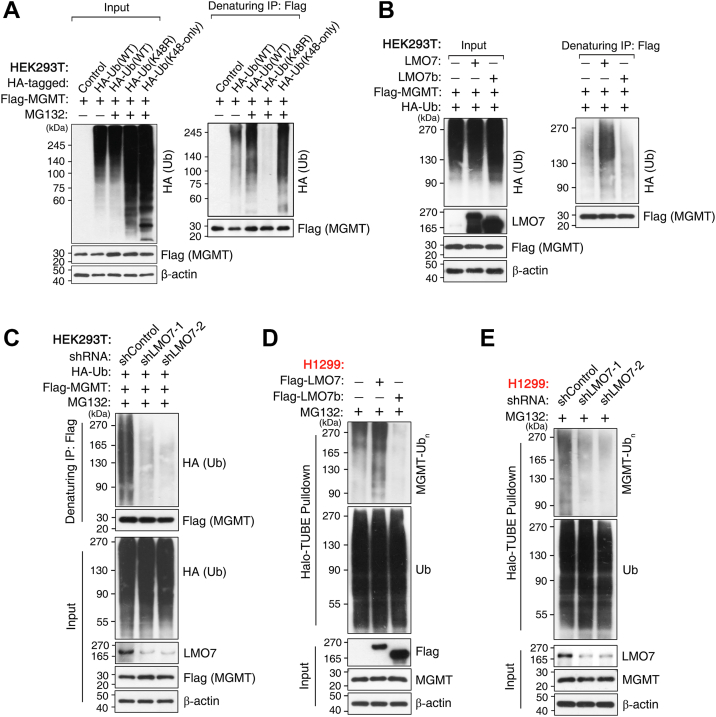
**LMO7 promotes K48-linked polyubiquitination of MGMT**. *A*, HEK293T cells expressing FLAG-MGMT were cotransfected with HA-ubiquitin WT (HA-Ub WT) or lysine-specific mutants (K48R or K48-only) and treated with MG132 (5 μM, overnight); MGMT ubiquitination was examined under denaturing conditions by immunoprecipitation (IP) with anti-FLAG M2-agarose beads followed by Western blotting (WB) with anti-HA. *B*, overexpression of LMO7-WT, but not the F-box–deficient mutant LMO7b, increased MGMT ubiquitination in HEK293T cells coexpressing FLAG-MGMT and HA-Ub after MG132 treatment (5 μM, overnight). *C*, LMO7 knockdown using two independent shRNAs reduced MGMT ubiquitination in HEK293T cells treated with MG132 (5 μM, overnight); knockdown efficiency was confirmed by WB. *D*, in H1299 cells, LMO7-WT, but not LMO7b, enhanced K48-linked MGMT ubiquitination detected using Halo-TUBE beads (K48-specific) under denaturing conditions following MG132 treatment (5 μM, overnight). *E*, LMO7 knockdown diminished K48-linked MGMT ubiquitination in H1299 cells, analyzed as in (*D*). Data are representative of n ≥ 3 biological replicates. HA, hemagglutinin; HEK293T, human embryonic kidney 293T cell line; LMO7, LIM domain only 7; MGMT, *O*^6^-methylguanine-DNA methyltransferase.

To determine the ubiquitin linkage on MGMT, HEK293T cells were cotransfected with FLAG-MGMT and HA-tagged ubiquitin constructs, including HA-Ub(WT), HA-Ub(K48R), and HA-Ub(K48-only). After MG132 treatment, denaturing IP revealed the accumulation of ubiquitinated MGMT species. MGMT ubiquitination was enhanced in cells expressing HA-Ub(K48-only) but was abolished with HA-Ub(K48R), indicating K48-linked polyubiquitination of MGMT (Fig. 2*A*). We next investigated whether LMO7 regulates MGMT ubiquitination. Overexpression of LMO7-WT increased MGMT ubiquitination in HEK293T cells cotransfected with FLAG-MGMT and HA-Ub(WT), whereas the ΔF-box mutant LMO7b did not (Fig. 2*B*). Conversely, shRNA-mediated depletion of LMO7 decreased MGMT ubiquitination (Fig. 2*C*).

To validate these observations in NSCLC cells and confirm K48 linkage specificity, denaturing Halo-tandem ubiquitin-binding entity (TUBE) (K48-specific) pull-down assays were performed in H1299 cells. LMO7-WT, but not LMO7b, increased K48-linked MGMT polyubiquitination (Fig. 2*D*), whereas LMO7 knockdown decreased this modification (Fig. 2*E*). Collectively, these results indicate that LMO7 promotes K48-linked polyubiquitination of MGMT, and the F-box–containing region is required for this activity.

### LMO7 regulates MGMT protein stability

**Figure 3:**
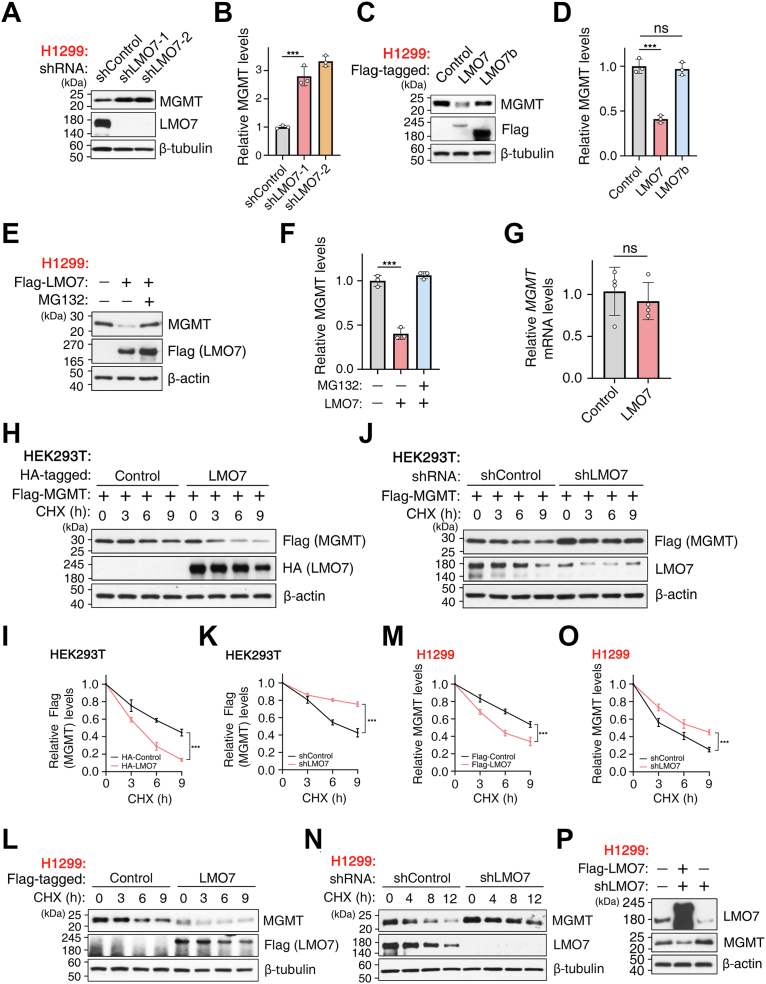
**LMO7 regulates MGMT protein stability**. *A*, WB of endogenous MGMT in H1299 cells transduced with shControl or two independent LMO7 shRNAs (#1, #2). *B*, quantification of MGMT levels from (*A*). *C*, WB of endogenous MGMT in H1299 cells expressing FLAG-LMO7-WT, the F-box–deficient mutant LMO7b, or vector control. *D*, quantification from (*C*). *E*, WB showing that LMO7 overexpression decreases MGMT levels, which are restored by MG132 cotreatment (5 μM, overnight). *F*, quantification from (*E*). *G*, qRT–PCR analysis showing that LMO7 overexpression does not significantly alter MGMT mRNA. *H*, cycloheximide (CHX, 180 μg/ml) chase in HEK293T cells stably expressing FLAG-MGMT, showing accelerated degradation with HA-LMO7; samples were collected at the indicated times and analyzed by WB. *I*, quantification from (*H*). *J*, CHX (180 μg/ml) chase in HEK293T cells showing prolonged FLAG-MGMT stability upon LMO7 knockdown. *K*, quantification from (*J*). *L*, CHX (180 μg/ml) chase in H1299 cells showing reduced half-life of endogenous MGMT upon LMO7 overexpression. *M*, quantification from (*L*). *N*, CHX (180 μg/ml) chase in H1299 cells showing stabilization of endogenous MGMT after LMO7 knockdown. *O*, quantification from (*N*). *P*, rescue experiment showing that re-expression of FLAG-LMO7 restores MGMT levels in shLMO7 H1299 cells. Data are representative of n ≥ 3 biological replicates. Error bars indicate mean ± SD. ∗∗∗*p* < 0.001 (unpaired, two-tailed Student’s *t* test). HA, hemagglutinin; HEK293T, human embryonic kidney 293T cell line; LMO7, LIM domain only 7; MGMT, *O*^6^-methylguanine-DNA methyltransferase; NS, not significant; qRT–PCR, quantitative RT–PCR; WB, Western blotting.

To determine whether LMO7 regulates endogenous MGMT protein levels, LMO7 expression was modulated in H1299 LUAD cells. Knockdown of LMO7 using two independent lentiviral shRNAs increased endogenous MGMT protein levels (Fig. 3*A*), and quantification confirmed this increase (Fig. 3*B*). In contrast, overexpression of LMO7-WT reduced MGMT protein levels, whereas the F-box deletion mutant (LMO7b) did not (Fig. 3, *C* and *D*). MG132 cotreatment reversed the LMO7-WT–induced reduction of MGMT (Fig. 3, *E* and *F*). LMO7 overexpression did not alter MGMT mRNA levels (Fig. 3*G*), indicating post-transcriptional regulation.

Consistent with these observations, similar regulatory effects were observed in A549 cells. LMO7 overexpression reduced MGMT, and MG132 restored MGMT protein levels (Fig. S1, *B* and *C*), whereas LMO7 knockdown increased MGMT (Fig. S1, *D* and *E*). Cycloheximide (CHX) chase assays further indicated accelerated MGMT degradation with LMO7-WT, no detectable effect with LMO7b, and stabilization upon LMO7 knockdown (Fig. S1, *F*–*I*), supporting LMO7-dependent control of MGMT protein stability across NSCLC models.

To directly assess MGMT protein stability, CHX chase assays were performed to block *de novo* protein synthesis. In HEK293T cells stably expressing FLAG-MGMT, LMO7 overexpression accelerated MGMT degradation and reduced the half-life (Fig. 3, *H* and *I*), whereas LMO7 knockdown prolonged the half-life of FLAG-MGMT (Fig. 3, *J* and *K*). Endogenous MGMT stability was then examined in H1299 NSCLC cells. LMO7 overexpression reduced the half-life of endogenous MGMT (Fig. 3, *L* and *M*), whereas LMO7 knockdown prolonged it (Fig. 3, *N* and *O*). Re-expression of FLAG-LMO7 in shLMO7 H1299 cells reversed the MGMT increase caused by LMO7 knockdown (Fig. 3*P*), indicating that LMO7 promotes MGMT degradation through the ubiquitin–proteasome pathway. Collectively, these results indicate that LMO7 reduces MGMT protein stability in NSCLC cells.

### TMZ enhances LMO7-mediated MGMT ubiquitination and degradation

**Figure 4:**
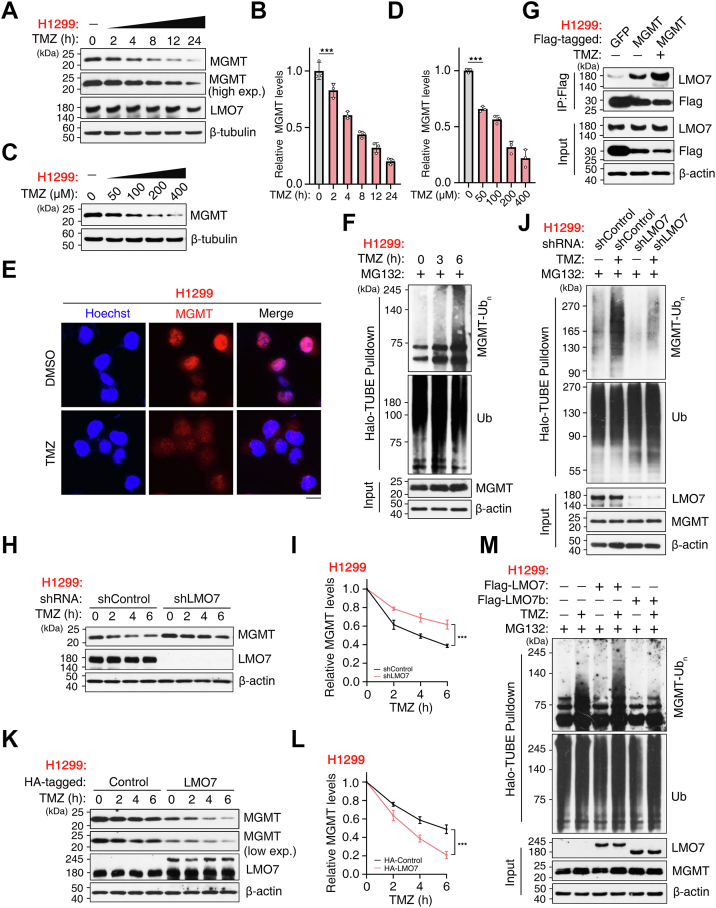
**Temozolomide (TMZ) enhances LMO7-mediated ubiquitination and degradation of MGMT**. *A*, WB showing time-dependent decrease of endogenous MGMT in H1299 cells treated with TMZ (100 μM) for the indicated times. *B*, quantification of relative MGMT levels from (*A*). *C*, WB showing dose-dependent reduction of MGMT in H1299 cells treated with increasing TMZ concentrations for 6 h. *D*, quantification from (*C*). *E*, immunofluorescence analysis showing MGMT loss in H1299 cells treated with TMZ (200 μM, 3 h); nuclei were counterstained with Hoechst. *F*, H1299 cells treated with 200 μM TMZ for the indicated times together with MG132 (20 μM, 6 h) were subjected to denaturing affinity purification using Halo-TUBE beads (K48-specific), and bound and input fractions were analyzed by WB for MGMT-linked polyubiquitin (MGMT-Ubn), ubiquitin, MGMT, and β-actin. *G*, co-IP of the LMO7–MGMT interaction in H1299 cells expressing FLAG-MGMT; cells received a TMZ pulse (300 μM, 20 min) and were immediately lysed for IP with anti-FLAG M2-agarose beads followed by WB. *H*, WB analysis of endogenous MGMT in H1299 cells transduced with shControl or shLMO7 and treated with TMZ (100 μM) for the indicated times. *I*, quantification from (*H*). *J*, H1299 cells transduced with shControl or shLMO7 were treated with TMZ (100 μM, 6 h) and MG132 (20 μM, 6 h) and analyzed under denaturing conditions using Halo-TUBE beads (K48 specific) to assess endogenous MGMT-Ubn. *K*, WB analysis of MGMT in H1299 cells overexpressing HA-LMO7 or control vector and treated with TMZ (100 μM) for the indicated times. *L*, quantification from (*K*). *M*, H1299 cells expressing FLAG-LMO7-WT or LMO7b were treated with TMZ (300 μM, 3 h) and MG132 (20 μM, 3 h), followed by denaturing Halo-TUBE enrichment to detect MGMT-Ubn. Data are representative of n ≥ 3 biological replicates. Error bars indicate mean ± SD. ∗∗∗*p* < 0.001 (unpaired, two-tailed Student’s *t* test). co-IP, coimmunoprecipitation; HA, hemagglutinin; LMO7, LIM domain only 7; MGMT, *O*^6^-methylguanine-DNA methyltransferase; TUBE, tandem ubiquitin-binding entity; WB, Western blotting.

We next examined whether TMZ alters MGMT protein stability in H1299 NSCLC cells. TMZ exposure caused a time-dependent decrease in endogenous MGMT protein levels (Fig. 4, *A* and *B*) and a dose-dependent reduction with increasing TMZ concentrations (Fig. 4, *C* and *D*). Immunofluorescence staining further indicated TMZ-associated loss of MGMT signal without an obvious change in subcellular localization (Fig. 4*E*).

To assess whether TMZ-induced MGMT reduction is coupled to ubiquitination, H1299 cells were treated with TMZ and MG132 and analyzed under denaturing conditions. Halo-TUBE enrichment revealed increased K48-linked MGMT polyubiquitination after TMZ treatment (Fig. 4*F*). We then tested whether TMZ modulates the association between LMO7 and MGMT. A short TMZ pulse increased the interaction between endogenous LMO7 and MGMT (Fig. 4*G*). Consistently, supplementary co-IP analysis indicated that a TMZ pulse strengthened the LMO7–MGMT interaction for MGMT-WT but not for the catalytically inactive MGMT-C145A mutant (Fig. S2, *A* and *B*), suggesting preferential association of LMO7 with catalytically engaged MGMT.

To determine whether LMO7 contributes to TMZ-induced MGMT ubiquitination and degradation, LMO7 was depleted or overexpressed during TMZ treatment. LMO7 knockdown attenuated the TMZ-induced decrease of endogenous MGMT (Fig. 4, *H* and *I*) and reduced K48-linked MGMT polyubiquitination under denaturing conditions (Fig. 4*J*). Conversely, LMO7-WT overexpression enhanced TMZ-induced MGMT degradation (Fig. 4, *K* and *L*), whereas the ΔF-box mutant LMO7b did not promote TMZ-induced MGMT ubiquitination (Fig. 4*M*). Collectively, these results indicate that TMZ strengthens the LMO7–MGMT interaction and enhances LMO7-dependent K48-linked polyubiquitination and proteasomal degradation of MGMT.

### LMO7 expression correlates with patient prognosis and sensitizes NSCLC cells to TMZ

**Figure 5:**
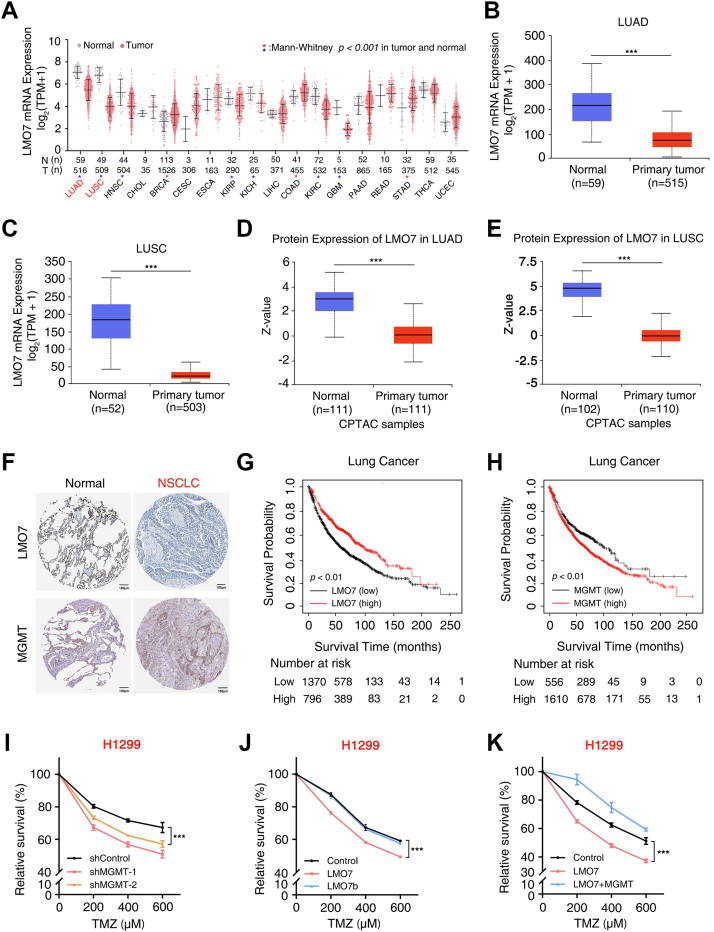
**LMO7 expression correlates with NSCLC patient prognosis and modulates TMZ sensitivity**. *A*, pan-cancer analysis of LMO7 mRNA expression across TCGA tumor and matched normal tissues shown as log2-transformed TPM values. *Blue asterisks* indicate lower LMO7 expression in tumors, and *red asterisks* indicate higher LMO7 expression in tumors. *B* and *C*, LMO7 mRNA expression in lung adenocarcinoma (LUAD) and lung squamous cell carcinoma (LUSC), comparing normal and primary tumors (TCGA *via* UALCAN). *D* and *E*, LMO7 protein expression in LUAD and LUSC, comparing normal and primary tumors (CPTAC *via* UALCAN; *z*-score). *F*, immunohistochemistry of LMO7 and MGMT in normal lung and NSCLC tissues (Human Protein Atlas). *G* and *H*, Kaplan–Meier overall survival curves for lung cancer patients stratified by high *versus* low LMO7 or MGMT expression; numbers at risk are indicated. *I*, cell viability of H1299 cells transduced with shControl or MGMT-specific shRNAs (#1, #2) treated with TMZ at the indicated concentrations. *J*, cell viability of H1299 cells expressing FLAG-LMO7-WT or LMO7b treated with TMZ. *K*, cell viability of H1299 cells expressing LMO7 alone or coexpressing LMO7 and MGMT treated with TMZ. Data are representative of n ≥ 3 biological replicates. Error bars indicate mean ± SD. ∗∗∗*p* < 0.001 (unpaired, two-tailed Student’s *t* test).

To evaluate the clinical relevance of LMO7 in NSCLC, publicly available transcriptomic and proteomic datasets were analyzed. LMO7 mRNA expression was reduced in both LUAD and lung squamous cell carcinoma relative to normal lung tissues in The Cancer Genome Atlas datasets (Fig. 5, *A*–*C*). Consistently, Clinical Proteomic Tumor Analysis Consortium proteomic profiles showed lower LMO7 protein levels in NSCLC tumors (Fig. 5, *D* and *E*). Immunohistochemistry data from the Human Protein Atlas further supported reduced LMO7 staining in NSCLC tumor sections and higher MGMT staining in tumors compared with adjacent normal lung tissue (Fig. 5*F*). Kaplan–Meier analysis indicated that low LMO7 expression was associated with reduced OS (Fig. 5*G*), whereas high MGMT expression correlated with reduced OS (Fig. 5*H*).

To test whether LMO7-dependent regulation of MGMT affects TMZ response, cell viability assays were performed in NSCLC cells. In H1299 cells, MGMT knockdown by two independent shRNAs was confirmed and maintained during the TMZ time course (Fig. S3, *A* and *B*), and MGMT depletion increased TMZ sensitivity (Fig. 5*I*). LMO7-WT, but not the F-box–deficient mutant LMO7b, sensitized H1299 cells to TMZ (Fig. 5*J*). Re-expression of MGMT reversed the TMZ-sensitizing effect of LMO7 (Fig. 5*K*), indicating that MGMT restoration counteracts LMO7-dependent TMZ sensitization. Similar trends were observed in A549 cells, where MGMT knockdown increased TMZ sensitivity (Fig. S3*C*), LMO7-WT but not LMO7b increased TMZ sensitivity (Fig. S3*D*), and MGMT re-expression reversed the effect (Fig. S3*E*). Collectively, these results indicate that reduced LMO7 expression is associated with poorer patient prognosis and that LMO7 enhances TMZ responsiveness in NSCLC cells through MGMT-dependent mechanisms.

## Discussion

**Figure 6:**
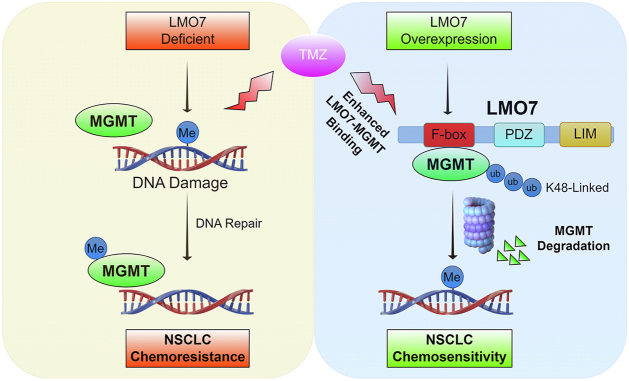
**Schematic model illustrating how LMO7 enhances TMZ sensitivity by promoting MGMT degradation**. In NSCLC cells with low LMO7 expression, MGMT remains stable and repairs TMZ-induced *O*^6^-methylguanine lesions, conferring resistance. When LMO7 is abundant, it promotes K48-linked polyubiquitination of MGMT *via* an associated E3 ubiquitination machinery and its proteasome-mediated degradation; TMZ-induced DNA damage further enhances the LMO7–MGMT interaction, accelerating MGMT loss and thereby increasing TMZ sensitivity. CPTAC, Clinical Proteomic Tumor Analysis Consortium; LMO7, LIM domain only 7; NSCLC, non–small cell lung cancer; TCGA, The Cancer Genome Atlas; TMZ, temozolomide; TPM, transcripts per million; UALCAN, The University of Alabama at Birmingham Cancer data analysis portal.

Here, we identify the LMO7–MGMT axis as an E3 ligase–linked regulator of TMZ sensitivity in NSCLC. LMO7 binds MGMT and promotes K48-linked polyubiquitination and proteasomal degradation of MGMT, reducing MGMT protein stability (Figure 1, Figure 2, Figure 3). TMZ exposure further strengthens the LMO7–MGMT interaction and increases K48-linked ubiquitination of MGMT, and genetic manipulation of LMO7 alters the extent of TMZ-induced MGMT loss (Fig. 4). Public datasets associate low LMO7 expression and high MGMT expression with shorter OS, whereas MGMT re-expression counteracts LMO7-mediated TMZ sensitization (Fig. 5). Together, these results indicate that an LMO7-mediated, ubiquitin-dependent mechanism regulates MGMT protein stability and is associated with TMZ sensitivity in NSCLC (Fig. 6).

A key mechanistic advance from this study is that LMO7 promotes ubiquitin-dependent proteasomal degradation of MGMT, coupling MGMT protein stability with TMZ sensitivity in NSCLC. This post-translational regulation is important because MGMT is widely viewed as a baseline determinant of alkylator response, yet the mechanisms that regulate MGMT protein levels during treatment remain incompletely understood in NSCLC, where TMZ has been explored for brain metastases, and resistance is common (19, 54, 55). Previous studies have focused on transcriptional and epigenetic mechanisms regulating MGMT mRNA expression as well as clinical associations with TMZ response (56, 57). In parallel, post-translational regulation of MGMT, including ubiquitin-dependent proteasomal degradation, is associated with increased alkylator sensitivity upon reduction of MGMT protein levels (41). However, the ubiquitin-dependent regulation of MGMT protein in NSCLC remains incompletely understood. Here, we show that LMO7 binds MGMT and promotes K48-linked polyubiquitination and proteasomal degradation of MGMT (Figure 1, Figure 2, Figure 3), thereby limiting MGMT protein levels in NSCLC cells. Domain mapping indicates that the F-box region is required for MGMT binding and ubiquitination under the tested conditions (Figs. 1 and 2), consistent with an SCF/CRL1 substrate–adaptor mechanism (58, 59) in which LMO7 couples MGMT recognition to ubiquitin transfer and proteasomal degradation.

This study reveals that TMZ exposure enhances the LMO7–MGMT axis by increasing the LMO7–MGMT interaction and promoting ubiquitin-dependent proteasomal degradation of MGMT, suggesting a drug-responsive amplification circuit. Although elevated MGMT protein levels are widely associated with TMZ resistance across tumor types and are most often discussed in the context of baseline expression and promoter regulation (60, 61, 62), whether TMZ exposure can engage a post-translational mechanism that promotes MGMT proteasomal degradation in NSCLC remains poorly defined. Here, we show that TMZ increases K48-linked polyubiquitination of MGMT and enhances the LMO7–MGMT interaction (Fig. 4). Manipulation of LMO7 correspondingly modulates TMZ-induced MGMT degradation, suggesting an LMO7-dependent component of MGMT loss during TMZ exposure. Notably, TMZ increases the LMO7–MGMT interaction to a greater extent with MGMT-WT than with the catalytically inactive MGMT-C145A mutant (Fig. 4 and Fig. S2), consistent with a contribution of MGMT catalytic competence to TMZ-enhanced binding. MGMT-C145A is catalytically inactive and cannot undergo Cys145 alkylation during suicide repair, so this comparison serves as an indirect readout rather than a direct measurement of MGMT alkylation status. Direct biochemical measurement of MGMT alkylation states during TMZ exposure would directly test this interpretation. These observations are consistent with a working model in which MGMT catalytic engagement, together with conformational and/or binding-state changes, increases accessibility for LMO7 association, thereby promoting LMO7-dependent ubiquitination and MGMT proteasomal degradation during TMZ exposure.

Clinically, our results link LMO7–MGMT status with survival and MGMT-dependent TMZ response in NSCLC. In public cohorts, low LMO7 expression and high MGMT levels are each associated with shorter OS (Fig. 5, *G* and *H*), and MGMT restoration counteracts LMO7-dependent TMZ sensitization in NSCLC cells (Fig. 5*K*). These data suggest that loss of LMO7-dependent MGMT degradation can promote MGMT accumulation and reduce TMZ sensitivity, a relationship that may be especially relevant in NSCLC brain metastases, where TMZ has been explored for its ability to cross the blood–brain barrier and exert central nervous system activity (63, 64). Together, our results support evaluating LMO7 and MGMT as a stratification framework that may help identify NSCLC subsets in which MGMT-dependent mechanisms modulate TMZ sensitivity. In addition, prior work reports that reduced LMO7 levels are associated with decreased cisplatin sensitivity in NSCLC (53), motivating tests of whether LMO7 status may help identify tumors that benefit from TMZ-based therapy alone or in combination with platinum agents, including in NSCLC brain metastases, where treatment options remain limited (54, 55, 65). On this basis, strategies that strengthen the LMO7–MGMT interface or phenocopy the LMO7-dependent MGMT degradation program may reduce MGMT protein level and thereby increase TMZ sensitivity, supporting translational testing in disease-relevant models and clinical specimens.

In summary, this study characterizes an LMO7-dependent ubiquitin axis that promotes K48-linked polyubiquitination and proteasomal degradation of MGMT, thereby increasing TMZ sensitivity in NSCLC. TMZ further strengthens the LMO7–MGMT interaction in an MGMT catalytic activity–dependent manner, suggesting a positive feedback loop that promotes MGMT clearance during TMZ exposure. In public cohorts, low LMO7 expression and high MGMT expression are each associated with shorter OS, and rescue experiments confirm that MGMT is required for LMO7-dependent TMZ sensitization. This work relies primarily on cellular models and public datasets, and validation in disease-relevant *in vivo* models, including NSCLC brain metastasis models, as well as in patient-derived specimens, will be needed to evaluate the clinical relevance of the LMO7–MGMT axis. Future studies identifying upstream regulators of LMO7 and evaluating approaches that enhance MGMT degradation through the LMO7–MGMT interface may inform strategies to increase TMZ efficacy in NSCLC.

## Experimental procedures

### Cell culture

HEK293T cells were obtained from the American Type Culture Collection, and H1299 cells were purchased from the Cell Bank of the Chinese Academy of Sciences. Cells were maintained in Dulbecco’s modified Eagle's medium (11965092, Gibco) supplemented with 10% fetal bovine serum (10270-106, Gibco) and 1% penicillin–streptomycin (100 U/ml penicillin and 100 μg/ml streptomycin; Gibco). All cultures were incubated at 37 °C in a humidified atmosphere containing 5% CO_2_. Mycoplasma contamination was routinely tested to ensure experimental reliability.

### Antibodies and reagents

The following primary antibodies were used: anti-MGMT (17195-1-AP, Proteintech), anti-HA (51064-2-AP, Proteintech), anti-LMO7 (ab224113, Abcam), anti-FLAG (F3165, Sigma–Aldrich), and anti-ubiquitin (sc-8017, Santa Cruz Biotechnology). Horseradish peroxidase (HRP)–conjugated anti-β-actin (A00730, GenScript) and anti-β-tubulin (E021043, EarthOx) were used as loading controls. HRP-conjugated secondary antibodies included anti-rabbit IgG (SA00001-2) and anti-mouse IgG (SA00001-1) (Proteintech). TMZ (S1237) was purchased from Selleck. CHX (180 μg/ml) was obtained from CST (2112S). The protease and phosphatase inhibitor cocktail was from Beyotime (P1051).

### Western blotting

Cells were washed twice with ice-cold PBS and lysed directly in 2× Laemmli sample buffer containing 2 mM DTT. Lysates were denatured by boiling at 98 °C for 10 min. Proteins were separated by SDS-PAGE and transferred to polyvinylidene fluoride membranes (Millipore). Membranes were blocked with 10% (w/v) nonfat milk in Tris-buffered saline with Tween-20 (TBST) buffer (50 mM Tris–HCl, pH 7.5, 150 mM NaCl, and 0.1% Tween-20) for 1 h at room temperature. Membranes were incubated with primary antibodies overnight at 4 °C, followed by five washes with TBST. HRP-conjugated secondary antibodies were applied for 1 h at room temperature. Blots were developed with enhanced chemiluminescence reagents, and densitometric analysis was performed with ImageJ (National Institutes of Health). All Western blotting (WB) data represent at least three independent experiments (n ≥ 3).

### Plasmid constructs, transfection, and lentiviral infection

The pcDNA3.1-His-LMO7 plasmid was obtained from Youbio Biological Technology. Full-length human LMO7 complementary DNA (cDNA) was used as a template to generate specific domain deletion mutants—such as LMO7b (ΔF-box), ΔPDZ, and ΔLIM—by site-directed mutagenesis. These LMO7 variants were subsequently subcloned into either the pCMV-FLAG or pHAGE-FLAG vector for transient or stable expression, respectively. Transient transfections were carried out using Lipo8000 Transfection Reagent (C0533, Beyotime) following the manufacturer’s instructions.

For lentivirus production, HEK293T cells were cotransfected with either pLKO.1 vectors (for shRNA expression) or pHAGE vectors (for LMO7 overexpression), together with standard lentiviral packaging plasmids. At 72 h post-transfection, viral supernatants were collected, filtered through 0.45 μm membranes, and used to infect target cells in the presence of 6 μg/ml polybrene for 12 h. Transduction efficiency was routinely validated by WB.

The target sequences for the shRNA against LMO7 and MGMT were shLMO7-1: 5′-AAATGATAACAAAGACCCAGG-3′; shLMO7-2: 5′-TTCTATGTAATCTAAAGAGGC-3′; shMGMT-1: 5′-TGCCACTTCCTTTAATACAGC-3′; shMGMT-2: 5′-GGCTGCTAATTGCTGGTAAGA-3′.

### MBP pull-down assay

MBP-tagged LMO7 constructs (MBP-LMO7), MBP-LMO7b, MBP alone, and FLAG-tagged MGMT, all expressed in Rosetta (DE3) *Escherichia coli* (Merck), were used for pull-down assays. Bacterial cultures harboring the respective expression plasmids were grown at 37 °C until an absorbance of 0.8 at 600 nm was reached. Protein expression was then induced with 0.5 mM IPTG, followed by overnight incubation at 16 °C. Bacterial pellets were collected by centrifugation at 8000*g* for 15 min at 4 °C and resuspended in lysis buffer as previously described. Lysates containing MBP-LMO7, MBP-LMO7b, or MBP alone were incubated with Amylose Resin (E8021S, New England Biolabs) at 4 °C for 12 h under constant rotation to allow fusion protein binding. After three washes with the wash buffer (as previously described), the resin was incubated with bacterial lysates containing FLAG-MGMT at 4 °C for 2 h. Following three additional washes, bound proteins were eluted by boiling in Laemmli sample buffer and analyzed by WB.

### Immunoprecipitation

Cells were harvested and lysed by sonication on ice in IP lysis buffer (150 mM NaCl, 50 mM Tris–HCl, pH 7.9, 1% Triton X-100, 10% glycerol, and 2 mM DTT), supplemented with protease and phosphatase inhibitor cocktail (P1051, Beyotime). Lysates were clarified by centrifugation at 13,000*g* for 10 min at 4 °C. The supernatants were then incubated overnight at 4 °C with gentle rotation using antibody-conjugated agarose beads, specifically anti-FLAG M2-agarose beads (A2220, Sigma) or HA-agarose beads (SC-7392AC, Santa Cruz). Beads were pelleted by brief centrifugation (1000*g*, 30 s) and washed five times with wash buffer (identical to the lysis buffer but without inhibitors). Bound proteins were eluted by boiling in Laemmli sample buffer at 98 °C for 5 min.

### Mass spectrometry

In H1299 cells stably expressing FLAG-tagged LMO7, protein complexes were isolated *via* immunoaffinity purification using anti-FLAG M2 affinity beads. After extensive washing, bound proteins were eluted with FLAG peptide (F3290, Sigma). The eluted samples were resolved by SDS-PAGE, visualized through silver staining, and then analyzed by LC–MS/MS. Mass spectrometric analysis was conducted using an Easy-nLC 1200 system (Thermo Fisher Scientific) connected to a Dr Maisch GmbH C18 column (75 μm × 150 mm, 3 μm). Data were acquired in data-dependent acquisition mode on a Q Exactive HF-X mass spectrometer (Thermo Fisher Scientific). Raw mass spectrometry data were searched against the UniProt human reference proteome database (UP000005640; 81791-20230317.fasta).

### CHX chase assay

Protein stability was assessed using a CHX chase assay. Cells were treated with 180 μg/ml CHX (2112S, CST) to inhibit *de novo* protein synthesis. Cells were harvested at the indicated time points and analyzed by WB using the specified antibodies.

### RNA extraction and quantitative RT–PCR

Total RNA was extracted using the RNAeasy RNA Isolation Kit (R0026, Beyotime) according to the manufacturer’s instructions. RNA concentration and purity were determined using a NanoDrop spectrophotometer (Thermo Fisher Scientific) by measuring absorbance at 260 nm and calculating absorbance at 260 nm/absorbance at 280 nm and absorbance at 260 nm/absorbance at 230 nm ratios. First-strand cDNA synthesis was performed using the BeyoFast cDNA Synthesis Kit (D7180M, Beyotime), followed by quantitative PCR (qPCR) using the BeyoFast SYBR Green qPCR Mix (D7260, Beyotime) according to the manufacturer’s instructions. Gene expression levels were normalized to β-actin as an internal control. The primers used for qRT–PCR were as follows: *MGMT*, forward primer, 5′-TTT TCC AGC AAG AGT CGT TCA C-3′, and reverse primer, 5′-GGG ACA GGA TTG CCT CTC AT-3′; *β-actin*, forward primer, 5′-TCA TCA CTA TTG GCA ACG AGC GGT TC-3′, and reverse primer, 5′-TAC CAC CAG ACA GCA CTG TGT TGG CA-3′.

### Affinity purification of K48-linked polyubiquitinated proteins using Halo-TUBE

A Halo-TUBE affinity purification approach was used to isolate K48-linked polyubiquitinated proteins. Following TMZ treatment, cells were harvested and lysed by boiling in TBS containing 1% SDS for 15 min. Lysates were diluted with IP lysis buffer (described in the *Immunoprecipitation* section) to a final SDS concentration of 0.1% (w/v). Cell extracts were incubated with 10 μl of HaloLink resin (G1915, Promega) prebound with 10 μg of purified Halo-TUBE protein per sample. Incubation was carried out overnight at 4 °C with gentle rotation. Afterward, the resin was washed five times with wash buffer (as described in the *Immunoprecipitation* section). Bound proteins were eluted by boiling in Laemmli sample buffer for 10 min and analyzed by WB.

### Cell viability assay

Cell viability was assessed using the Cell Counting Kit-8 (Beyotime). Cells were seeded into 96-well plates and incubated overnight (16–20 h). They were then treated with TMZ or vehicle control for 48 h. After treatment, 10 μl of Cell Counting Kit-8 reagent was added to each well, and the plate was incubated at 37 °C for 2 h. Absorbance at 450 nm was measured using a microplate reader.

### Immunofluorescence assay

Cells were seeded on sterile round glass coverslips in 12-well plates. After 24 h, cells were treated with TMZ for 3 h, then fixed in 4% (w/v) paraformaldehyde in PBS for 30 min at room temperature. Fixed cells were permeabilized with 0.5% (v/v) Triton X-100 in PBS for 20 min, followed by three washes with TBST (as described above). Cells were blocked in 5% bovine serum albumin for 1 h at room temperature and incubated with primary antibodies overnight at 4 °C with gentle shaking. The next day, cells were washed four times with TBST and incubated with fluorescently labeled secondary antibodies and Hoechst 33342 at 37 °C for 1 h in the dark. Coverslips were then mounted using antifade mounting medium (P0126, Beyotime), and images were captured using a fluorescence microscope (Olympus).

### Bioinformatics and statistical analysis

Densitometric quantification of WB signals was performed using ImageJ software. Graphs and statistical analyses were generated using GraphPad Prism 7.0 (GraphPad Software). Data are presented as mean ± SD from at least three independent experiments (n ≥ 3). Statistical significance between two groups was assessed using Student’s *t* test, whereas one-way ANOVA was used to compare multiple groups. A *p* value <0.05 was considered statistically significant (∗*p* < 0.05, ∗∗*p* < 0.01, and ∗∗∗*p* < 0.001); nonsignificant differences are indicated as ns.

Publicly available RNA-Seq data from The Cancer Genome Atlas *via* the University of Alabama at Birmingham Cancer data analysis portal (http://ualcan.path.uab.edu) and proteomics data from the Clinical Proteomic Tumor Analysis Consortium were analyzed to assess LMO7 expression. Kaplan–Meier survival analyses were conducted using the Kaplan–Meier Plotter database (http://kmplot.com/analysis/) for lung cancer patient datasets.

## Data availability

All data generated or analyzed during this study are included in the article.

## Supporting information

This article contains supporting information.

## Ethics statement

This study did not involve experiments with human participants or animals.

## Conflict of interest

The authors declare that they have no conflicts of interest with the contents of this article.
