# Supplementary material for: E3 ligase LMO7 enhances temozolomide sensitivity by promoting MGMT degradation in lung cancer
Source: J Biol Chem. 2026 Feb 26;302(4):111331. doi: 10.1016/j.jbc.2026.111331 (PMC13022618; doi:10.1016/j.jbc.2026.111331)
Supplement: Supplementary Material 2 [file mmc2.pdf]

SUPPLEMENTARY INFORMATION

E3 ligase LMO7 enhances temozolomide sensitivity by promoting MGMT degradation in lung cancer

Jiabing Li[1#], Xiaorong Feng[1#], Yunfang Deng[1#], Bei Chen[1], Lin Miao[1], Zhaohui Liu[1], Zhiming Sun[1], Lei Hu[1], Jinqiu Ma[1], Liyuan Zeng[1], Xiaolong Wang[1], Yu Zhao[1,2*]

[1]The National & Local Joint Engineering Laboratory of Animal Peptide Drug Development, College of Life Sciences, Hunan Normal University, Changsha, Hunan, 410081, China

[2]Peptide and Small Molecule Drug R&D Platform, Furong Laboratory, Hunan Normal University, Changsha, Hunan, 410081, China

* Corresponding author: Yu Zhao (smile_zhao@hotmail.com)

[#] These authors contributed equally to this work.

Running title: LMO7 enhances TMZ sensitivity in lung cancer

Keywords: LMO7; MGMT; E3 ligase; ubiquitination; NSCLC; temozolomide; chemoresistance

SUPPLEMENTARY FIGURE LEGEND

Supplementary Figure S1. LMO7 regulates MGMT protein level and stability in A549 cells. *A*, Endogenous co-immunoprecipitation (co-IP) using an anti-LMO7 antibody with IgG control in A549 cells, and immunoprecipitates and unbound fractions (flow-through) were analyzed by western blotting (WB) to detect endogenous LMO7 and MGMT. *B,* WB analysis showing that Flag-LMO7 overexpression reduces endogenous MGMT protein level in A549 cells; co-treatment with MG132 (5 μM, overnight) restores MGMT protein level. *C,* Quantification of MGMT protein levels from (*B*). *D,* WB showing that knockdown of LMO7 using two independent shRNAs (#1 and #2) increases MGMT protein level in A549 cells; knockdown efficiency was verified in the input WBs. *E,* Quantification from (*D*). *F,* Cycloheximide (CHX, 180 μg/mL) chase in A549 cells expressing Flag-LMO7-WT, the F-box–deficient mutant LMO7b, or vector control, showing accelerated MGMT degradation with LMO7-WT and no effect with LMO7b; samples were collected at the indicated times and analyzed by WB. *G,* Quantification from (*F*). *H,* CHX chase (180 μg/mL) showing stabilization of endogenous MGMT protein in A549 cells upon LMO7 knockdown using two independent shRNAs. *I,* Quantification from (*H*. Data are representative of $n \geq 3$ biological replicates. Error bars indicate mean ± SD. ***$p < 0.001$ (unpaired, two-tailed Student's t-test).

Supplementary Figure S2. MGMT catalytic state modulates the TMZ-enhanced LMO7–MGMT interaction. Co-IP analysis in HEK293T cells comparing the interaction between LMO7 and MGMT-WT versus the catalytically inactive MGMT-C145A mutant. Cells received a TMZ pulse (300 μM, 20 min) and were immediately lysed for Flag IP using anti-Flag M2-agarose beads, followed by WB to detect the indicated proteins. Data are representative of $n \geq 3$ biological replicates. *B,* Quantification from (*A)*. Data are representative of $n \geq 3$ biological replicates. Error bars indicate mean ± SD. ***$p < 0.001$ (unpaired, two-tailed Student's t-test)

Supplementary Figure S3. Validation of MGMT knockdown in H1299 cells and analysis of

LMO7-mediated modulation of TMZ sensitivity in A549 cells. *A,* WB validation of MGMT knockdown in H1299 cells transduced with shControl or two independent MGMT shRNAs. *B,* WB analysis of endogenous MGMT protein level in H1299 cells expressing shControl or shMGMT during continuous TMZ treatment (100 μM) for the indicated times. *C*, Cell viability of A549 cells transduced with shControl or two independent MGMT shRNAs and treated with the indicated concentrations of TMZ. *D,* A549 Cell viability of overexpressing Flag-LMO7 or the F-box–deficient mutant LMO7b treated with increasing concentrations of TMZ. *E,* Re-expression of MGMT restores TMZ resistance in A549 cells overexpressing LMO7. Data are representative of $n \geq 3$ biological replicates. Error bars indicate mean ± SD. ***$p < 0.001$ (unpaired, two-tailed Student's t-test).

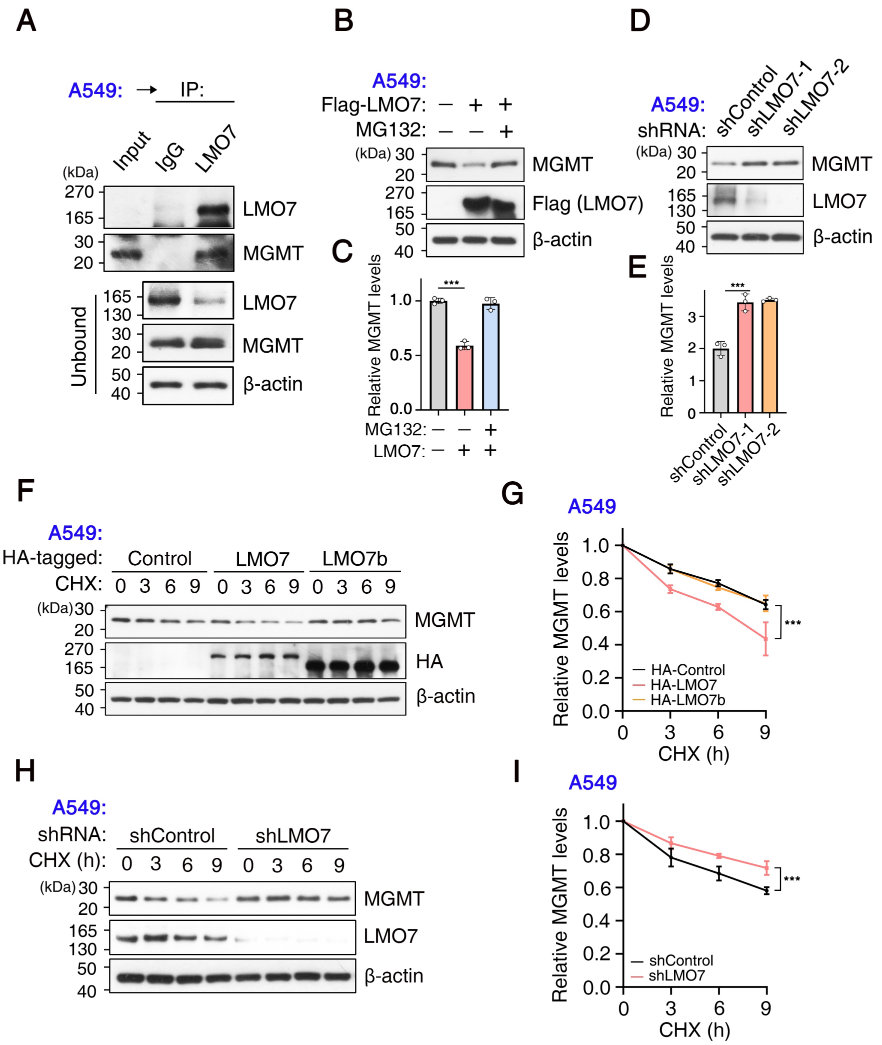
A
A549:
IP:
Input
IgG
LMO7
(kDa)
270
165
LMO7
30
20
MGMT
Unbound
165
130
LMO7
30
20
MGMT
50
40
β-actin
B
A549:
Flag-LMO7: − + +
MG132: − − +
(kDa) 30
20
MGMT
270
165
Flag (LMO7)
50
40
β-actin
C
Relative MGMT levels
***
MG132: − − +
LMO7: − + +
D
A549:
shRNA:
shControl
shLMO7-1
shLMO7-2
(kDa) 30
20
MGMT
165
130
LMO7
50
40
β-actin
E
Relative MGMT levels
***
shControl
shLMO7-1
shLMO7-2
F
A549:
HA-tagged: Control LMO7 LMO7b
CHX: 0 3 6 9 0 3 6 9 0 3 6 9
(kDa) 30
20
MGMT
270
165
HA
50
40
β-actin
G
A549
Relative MGMT levels
HA-Control
HA-LMO7
HA-LMO7b
***
CHX (h)
H
A549:
shRNA: shControl shLMO7
CHX (h): 0 3 6 9 0 3 6 9
(kDa) 30
20
MGMT
165
130
LMO7
50
40
β-actin
I
A549
Relative MGMT levels
shControl
shLMO7
***
CHX (h)

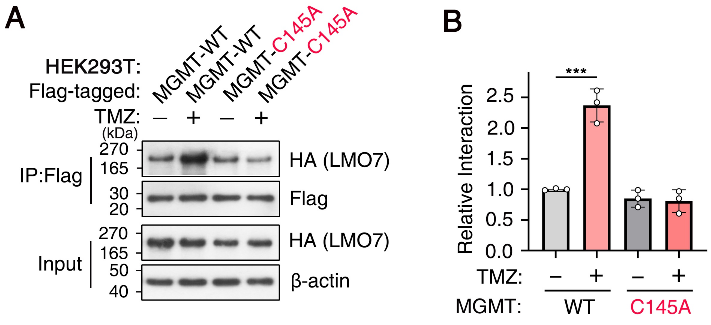
A
HEK293T:
Flag-tagged:
MGMT-WT
MGMT-WT
MGMT-C145A
MGMT-C145A
TMZ: – + – +
(kDa)
270
165
HA (LMO7)
IP:Flag
30
20
Flag
270
165
HA (LMO7)
Input
50
40
β-actin
B
***
Relative Interaction
2.5
2.0
1.5
1.0
0.5
0.0
TMZ: – + – +
MGMT: WT C145A

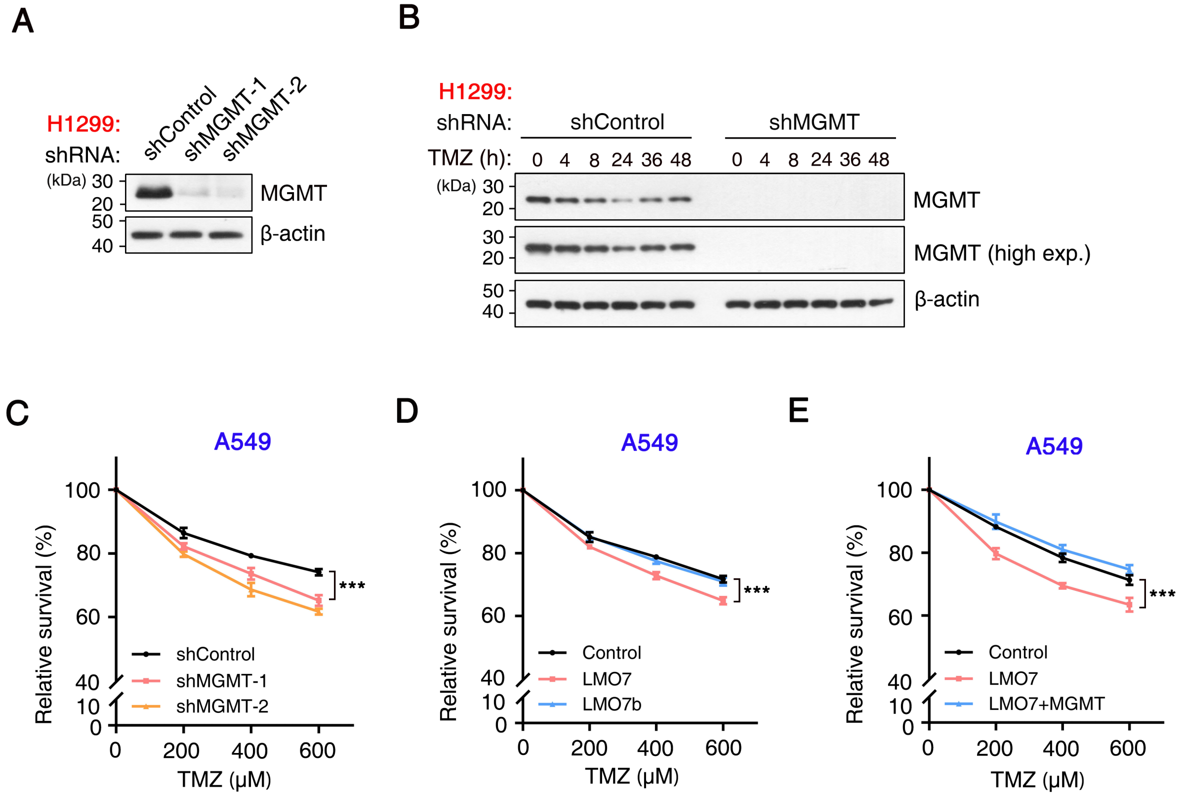
A
H1299:
shRNA:
shControl
shMGMT-1
shMGMT-2
(kDa) 30
20
50
40
MGMT
β-actin
B
H1299:
shRNA:
shControl
shMGMT
TMZ (h):
0 4 8 24 36 48
0 4 8 24 36 48
(kDa) 30
20
30
20
50
40
MGMT
MGMT (high exp.)
β-actin
C
A549
Relative survival (%)
100
80
60
40
10
0
shControl
shMGMT-1
shMGMT-2
***
0 200 400 600
TMZ (μM)
D
A549
Relative survival (%)
Control
LMO7
LMO7b
***
TMZ (μM)
E
A549
Relative survival (%)
Control
LMO7
LMO7+MGMT
***
TMZ (μM)
